# Thermodynamic and Kinetic Investigation of the Adsorption and Desorption of Trimethoprim and Its Main Metabolites in Mediterranean Crop Soils

**DOI:** 10.3390/molecules28010437

**Published:** 2023-01-03

**Authors:** Carmen Mejías, Juan Luis Santos, Julia Martín, Irene Aparicio, Esteban Alonso

**Affiliations:** Departamento de Química Analítica, Escuela Politécnica Superior, Universidad de Sevilla, C/Virgen de África, 7, E-41011 Seville, Spain

**Keywords:** adsorption, kinetic, antibiotics, trimethoprim, metabolites, agricultural soils, isotherms

## Abstract

The adsorption–desorption processes of organic pollutants into the soil are one of the main factors influencing their potential environmental risks and distribution in the environment. In the present work, the adsorption–desorption behavior of an antibiotic, trimethoprim (TMP), and two of its main metabolites, 3-desmethyltrimethoprim (DM-TMP) and 4-hydroxytrimethoprim (OH-TMP), were assessed in three Mediterranean agricultural soils with different physicochemical characteristics. Results showed that the adsorption kinetic is performed in two steps: external sorption and intraparticle diffusion. The adsorptions of the studied compounds in soils were similar and fitted to the three models but were better fitted to a linear model. In the case of DM-TMP and OH-TMP, their adsorptions were positively correlated with the soil organic matter. In addition, desorption was higher in less organic matter soil (from 1.3 to 30.9%). Furthermore, the desorptions measured for the TMP metabolites were lower than those measured in the case of TMP (from 2.0 and 4.0% for OH-TMP and DM-TMP, respectively, to 9.0% for TMP).

## 1. Introduction

The growing population in urban areas and the changes in living standards have led to an increase in water consumption and, as a result, an increase in the amount of treated wastewater and sewage sludge. One of the main disposal options for these products is their reuse in agricultural settings through their application to the soil (in the case of sludge) and crop irrigation (in the case of wastewater). However, the presence of organic environmental pollutants in wastewater and sludge is one of the major drawbacks of these practices.

Pharmaceutically active compounds (PhACs) are the emerging contaminants that have probably received the most scientific attention in recent years. Among them, antibiotics are the PhACs that generate a major concern because of their continuous consumption and discharge into the sewer system, biological activity against living organisms, and the increase of antibiotic-resistant bacteria [1]. The presence of antibiotics in agricultural soil, not only from sludge and wastewater reuse but also from animal manure, has been described in several papers [2,3,4]. For example, antibiotics, such as fluoroquinolones, sulfonamides, macrolides, or tetracyclines, were measured in agricultural soils at concentrations up to the mg per kilogram level [3,5,6,7,8]. These studies show trimethoprim (TMP), an antibiotic usually administered with sulfamethoxazole, as one of the most recurrent antibiotics measured in agricultural soil. After intake, 80% of TMP is still unaltered, while the other 20% is metabolized [9]. These metabolites are produced by demethylation of the parent compound (3-desmethyl-trimethoprim (DM-TMP), 65% of metabolized TMP, and 4-desmethyl-trimethoprim, 25% of metabolized TMP), and by its hydroxylation (4-hydroxy-trimethoprim (OH-TMP), 2% of metabolized TMP) [9]. These compounds could not only affect the soil organism but also contaminate surface water through runoff, groundwater by leaching, or even crops entering the food chain [2,10]. However, to date, there are no studies focused on the distribution and presence of these metabolites in soil, even though they may possess the same biological activity as the parent compound, or they could be present at higher concentrations or even more toxic than their parent compound [11].

The adsorption and desorption of TMP and its metabolites in the soil is one of the main issues controlling their occurrence, fate, and even potential ecotoxicological effects in the environment [1,2,10,12]. Among others, their adsorption in the soil can be produced by Van der Waals forces, hydrogen bodings, hydrophobic interactions, or surface complexes [1,10]. These interactions are governed by the physicochemical properties of the compound (p*K*_a_, polarity, and octanol–water partition coefficient) or of the soil (organic matter, pH, and exchangeable cation capacity) [10]. This shows the need to evaluate the adsorption and desorption of each compound case-by-case in relation to the soil characteristics to know its mobility and the potential influence on its environmental risks. However, despite the fact that the adsorption of several compounds has been previously evaluated (such as polycyclic aromatic hydrocarbons [13,14], pesticides [15,16], and even PhACs [17,18,19,20,21] including TMP), to the best of our knowledge, there are no previous studies about the adsorption or desorption of TMP metabolites in Mediterranean soils. In addition, most of the reported data is focused on the adsorption of PhACs into the soil, while there is a lack of information on their desorption behavior.

The objective of the present work was to study the adsorption and desorption behavior of TMP and two of its metabolites produced from different metabolic routes (DM-TMP and OH-TMP) in three Mediterranean soils with different physicochemical characteristics. Mediterranean soils distributed in the Mediterranean zone were selected.

## 2. Results and Discussion

### 2.1. Preliminary Experiments

Results obtained for the evaluation of the soil/solution ratio (Figure S1) show adsorption variations with the increase in the amount of the soil. The adsorption of the compounds slightly increases with the rise of the soil/solution ratio. The highest adsorption rates were obtained in the case of the soil/solution ratio of 0.4:1, being constant in a soil/solution ration of 0.5:1. This behavior could be explained by the increase in the number of soil active sites at higher soil amounts for the concentration of antibiotics used.

### 2.2. Adsorption Kinetics

**Figure 1:**
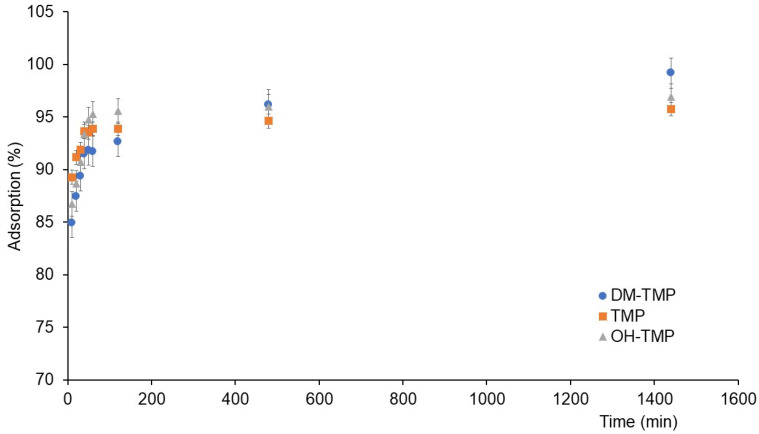
Adsorption kinetic of TMP and its metabolites.

Figure 1 shows the adsorption of studied compounds during kinetic experiments. The adsorption kinetics were evaluated by fitting the adsorption data to the pseudo-first-order (PFO) and pseudo-second-order (PSO) models. Moreover, intra-particle diffusion (IPD) was evaluated using the Weber–Morris model. The PFO and PSO models were assessed by plotting ln(*q*_e_ − *q*_t_) vs. t and t/*q*_t_ vs. t, respectively (Figures S2 and S3 in the Supplementary Materials). The results of the studied compounds’ adsorption kinetics are shown in Table 1. As can be seen, the adsorption kinetic data of the studied compounds were fitted to a PSO model (the *R*^2^ values for the PSO models were 0.999 in all cases, while the *R*^2^ values for the PFO models were from 0.4738 to 0.9496). This result could be due to the low initial concentration (1 mg/L) in relation to the abundant active sites in the soil, as was previously reported [22,23]; therefore, the sorption capacity of the studied compounds is controlled by the high availability of active sites in the soil. According to these results, the *q*_e-cal_ values were calculated using the PSO model. These *q*_e-cal_ values were 1.97, 2.06, and 2.03 μg/g for TMP, OH-TMP, and DM-TMP, respectively, which correspond with the amounts of the compounds adsorbed after 24 h of experiments (*q*_e-exp_) and show that 24 h is the equilibrium time for all studied compounds. The Weber–Morris IPD model was applied to determine the step (external diffusion, internal diffusion, or adsorption onto active sites) controlling the adsorption [23]. The two steps obtained using the IPD model corresponded to the two first steps in the adsorption of the studied compounds. The first step is controlled by surface sorption. Due to the abundance of active sites, this step occurs at a high rate. The second step, governed by interparticle diffusion, occurs when the active sites are depleted, and the molecule migrates inside the soil pores. The third step of the model corresponds to the equilibrium phase [23]. According to previously published papers about the adsorption of TMP [1,24,25] and other PhACs and their metabolites [17,18,19,20] in different soils and following the recommendations of the OECD [26], kinetic experiments were carried out from 0 to 1440 min (24 h). The studied period was enough to achieve equilibrium; however, the third step (slopes for the Weber–Morris plots (*k*_ip_) close to cero) was not viewable (these are usually viewable from 24 to several days). This has been described in the case of other contaminants whose kinetic was studied up to 24 h [27]. For all studied compounds, the C_1_ values were nonzero (Figure S4). These results indicate that the adsorption of the studied compounds could be involved in different steps [28,29,30].

Wu et al. (2009) [31] reported that the initial adsorption behavior could be described by Equation (1):

*q*_t_/*q*_ref_ = 1 − *R*_i_ [1 − (t/t_ref_)^1/2^] (1)

where t_ref_ is the highest time in the adsorption process, *q*_ref_ is the solid phase concentration at t_ref_ for an adsorption system, and *R*_i_ is the initial adsorption factor of the IPD model. Figure S5 shows the initial adsorption curves of TMP, OH-TMP, and DM-TMP on the IPD model according to Equation (1) (*q*_t_/*q*_ref_ vs. t/t_ref_). According to this model, *R*_i_ values for studied compounds were 0.07 (TMP), 0.11 (OH-TMP), and 0.14 (DM-TMP), which means that the initial adsorption (*q*_t_/*q*_ref_) has already reached 93% (TMP), 89% (OH-TMP), and 85% (DM-TMP). Then, the adsorption proceeds following the IPD mechanism. According to *k*_ip,1_ (Table 1), the external adsorption (first step) of DM -TMP and OH-TMP was faster than the adsorption of TMP, while in the second step, DM-TMP showed a higher IPD rate.

### 2.3. ATR FT-IR Characterization

**Figure 2:**
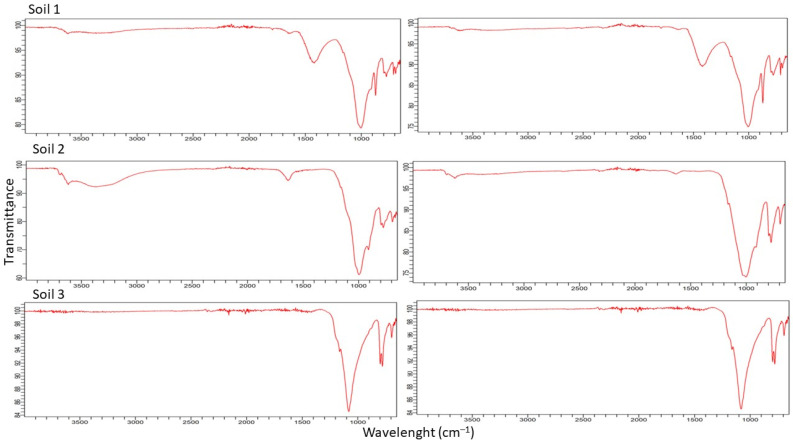
FT-IR transmittance spectra of three soils before (**left**) and after (**right**) the adsorption process.

The ATR FT-IR spectra of the soils did not show much difference between them (Figure 2). An asymmetric vibration band could correspond to Si–O (1090 cm^−1^), an asymmetric vibration band may be correlated with Si–OH (950 cm^−1^), a symmetric vibration band fits with Si–O (795 cm^−1^), and a bending vibration band is related to Si—O—Si (480 cm^−1^), which indicate the possible presence of silicates (SiO_2_) in the soils. Bands between 800 and 1260 cm^−1^ have been described as a superimposition of various SiO_2_ peaks and Si–OH bonding and peaks due to residual organic groups. After the adsorption test, no new peaks appeared in the FT-IR spectra, which could indicate that there was no covalent bond formed during the sorption process. The only slight difference is the intensity of some peaks. Thus, the adsorption would be mainly controlled by physical interaction.

### 2.4. Adsorption Isotherms

**Figure 3:**
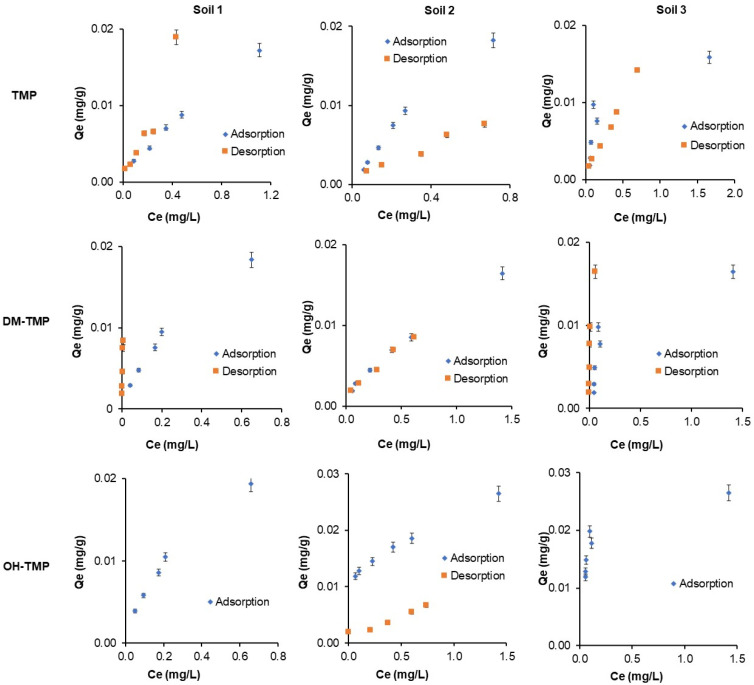
Adsorption and desorption isotherms of TMP and its metabolites in three Mediterranean soils.

Figure 3 shows the adsorption isotherms of TMP and its metabolites in three soils. The adsorption data were fitted with the Freundlich, Langmuir, and linear (Henry) models to clarify their adsorption characteristics. The parameters of the models are presented in Table 2. The data fitted well with all the Langmuir (0.993 > *R*^2^ > 0.740), Freundlich (0.997 > R^2^ > 0.721), and even better with the Henry (0.996 > *R*^2^ > 0.970) models for Soil 1 and 2. This tendency was not followed by Soil 3, where *R*^2^ decreases for the linear (0.734 > *R*^2^ > 0.717) and Freundlich (0.833 > *R*^2^ > 0.791) models but adjusted better with the Langmuir model (0.928 > *R*^2^ > 0.857). Therefore, Soil 3 has a less linear adsorption isotherm than Soils 1 and 2. With the increase of the TMP and metabolites concentrations, the adsorption was rapidly increased. Nevertheless, the saturation of the soils was not observed. This fact may be because of the low concentration range used in this work in an attempt to use real concentrations in the environment. Similar results were obtained by Zhang et al. (2014) and Kočárek et al. (2016) for TMP [21,24]. Moreover, considering the *K*_d_ values, the obtained linear correlation may imply a constant availability of the active sites in the soils.

Considering the surface heterogeneity parameter (1/*n*) obtained using the Freundlich model, its values were different from one for most of the studied compounds, which indicates that the sorption process could occur on the heterogeneous surface of the sorbent [32,33,34] and represents the adsorption strength between the pollutant and the soil. In general, the 1/*n* values lower than one indicate convex Freundlich isotherms, which represent stronger sorption, while 1/*n* values higher than one represent poorer sorption of the compound to the soil [32,33,34]. In this work, the weakest adsorption belongs to Soil 3 (1/*n* values from 2.72 to 5.26), followed by Soil 2 (1.27–3.61) and Soil 1 (1.29–1.58). These results could be related to the clay content of the studied soils (30.8, 19.9, and 16% for Soils 1, 2, and 3, respectively) and the higher adsorption sites in the finest particles of the soil. This behavior has been previously described for other soil contaminants [34]. The low values obtained for *K*_d_ (0.024–0.007 L/g), *K*_L_ (0.461–17.07 L/g), and *K*_F_ (0.013–0.028 L/g) confirm that compound retention is not strong and has a high tendency to be mobile in soils. The 1/*n* value has also been used to evaluate the type of adsorption process [35]. Similar results of isotherms parameters were obtained by Zhang et al. (2014) [21] for TMP. Furthermore, *K*_d_, *K*_L_, and *K*_F_ parameters increased for metabolites in comparison with TMP in all soils, resulting in higher adsorption of metabolites than TMP. Nevertheless, the *q*_max_ for metabolites increased in Soils 1 and 3 in comparison with TMP but decreased in Soil 2 in comparison with TMP, showing that TMP does not have a dependence on organic matter content in the soil.

### 2.5. Desorption Isotherms

Figure 3 shows the desorption isotherms of TMP and its metabolites in the three studied soils. No desorption was observed for OH-TMP in Soils 1 and 3. The desorption data obtained were fitted with the Freundlich, Langmuir, and linear models to clarify the desorption characteristics further. Desorption isotherms were fitted to the Langmuir (0.980 > *R*^2^ > 0.895), Freundlich (0.993 > *R*^2^ > 0.896), and Henry (0.996 > *R*^2^ > 0.800) models. The parameters of the models are listed in Table 2. The results reported by Rodríguez-López et al. (2022) [36] for TMP also satisfactorily fit the three models used: Freundlich, Langmuir, and linear. On the contrary to adsorption isotherms experiments, *K*_d_, *K*_F_, and *K*_L_ values must be interpreted, considering that higher scores indicate lower desorption and vice versa. Higher values were obtained for metabolites, indicating higher desorption in the case of TMP. The hydroxyl group probably has a positive effect on the adsorption process and a negative effect on the desorption process due to the possibility of forming new hydrogen bonds with the soil. Similar *K*_d_ results were obtained by Zhang et al. (2014) [21]. These results indicate that adsorbed compounds in the soil can be released into the environment, to groundwater by leaching, surface water by runoff, or even crops, entering the food chain and posing potential health and environmental risks.

### 2.6. Influence of Compounds Properties and Physicochemical Characteristics of the Soils on Compound Adsorption–Desorption Behavior

**Figure 4:**
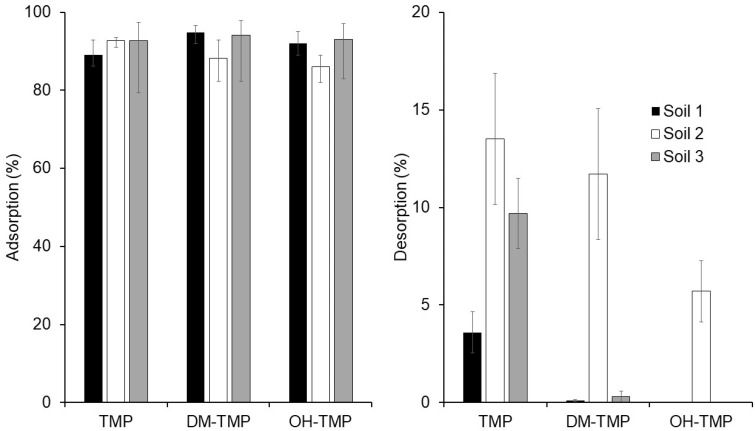
Adsorption and desorption percentage of TMP and its metabolites in three different soils.

Adsorption and desorption of TMP and its metabolites, measured as the mean value obtained in the determination of the adsorption and desorption isotherms, are shown in Figure 4. Globally, no significant differences were observed between the mean adsorptions measured in the studied soils. However, considering only the metabolites of TMP, the adsorptions measured in Soil 2 were significantly different from those measured in Soils 1 and 3 (Student’s *t*-test: *t*_cal_ = 6.39 and 12.89, in comparison with Soils 1 and 3, respectively; *t*_tab_ = 2.36, *p* < 0.05). In the case of TMP, the lowest adsorption was measured in Soil 1, while the adsorptions in Soils 2 and 3 were similar. Considering the desorption experiments, Soil 2 showed the highest desorption (mean 10.3%) of the studied compounds (Student’s *t*-test: *t*_cal_ = 8.098 and 6.558, in comparison with Soils 1 and 3, respectively; *t*_tab_ = 2.36, *p* < 0.05), followed by Soil 3 (3.3%) and Soil 1 (1.2%). These results could be related to the different physicochemical characteristics of the studied soils. According to the OECD [26], the key parameters of soil that play the most crucial role in the adsorption and desorption of organic pollutants are the pH of the soil for ionizable compounds, organic matter content, clay content, soil texture, and cation exchange capacity. The pH is important because antibiotics have p*K*_a_ values that determine speciation. The pH of the three different soils is very similar, so in this case, no significant differences are shown. The soil organic matter is fundamental for the interactions with hydrophobic pollutants. The presence of organic matter in three soils follows the order of 3 > 1 > 2, which correlates with the adsorption and desorption behavior of pollutants. However, the similar and low organic matter of the studied soils (lower than one in the case of Soils 1 and 2) does not allow for drawing clear conclusions. In order to establish a potential relationship between the measured adsorptions and desorption and the characteristics of the studied soils, statistical and correlational analyses were carried out. The correlational analysis was conducted considering the soils as cases and the physicochemical characteristics of the soils, and the adsorption and desorption of the studied compounds as variables. The correlation matrix is shown in Table S1. High positive correlations (higher than 0.60) were obtained between the organic matter and the adsorptions of compounds DM-TMP (0.60) and OH-TMP (0.84) considering the organic matter, while negative correlations (lower than −0.60) were obtained between the desorption of these compounds and the organic matter. On the contrary, no correlations were obtained between the organic matter and the adsorption and desorption of TMP. These results could be related to a different adsorption mechanism of the studied compounds, in which the hydroxyl group of the metabolites could play an important role in the interaction of these compounds with organic matter [17]. In the case of TMP, without a hydroxyl group, its adsorption behavior could be explained by considering its non-ionizable form [26]. These results, in the case of TMP, are in concordance with previous studies [1,21,24,26]. For example, Zhang et al. (2014) [21] reported that, based on p*K*_a_, TMP was in neutral form in neutral and basic mediums and obtained the highest adsorption affinity in acidic mediums. The pH of our three studied soils were 8.27, 8.21, and 8.06 in Soil 1, Soil 2, and Soil 3, respectively, supporting this hypothesis. These results are consistent with the study reported by Kočárek et al. (2016) [24], who have not found the dependence of TMP adsorption on the soil’s organic matter.

Considering the texture, the results obtained by the statistical analysis seem to be contradictory. For example, high negative correlations (<−0.68) were obtained in the case of the adsorption of TMP and fine sand, clay, and silt. However, the correlations obtained in the case of the desorption of this compound with fine sand, silt, and clay were negative, too (lower than −0.80). This could be due to the high adsorption measured for TMP in the studied soils. In the desorption experiments, high negative correlations were obtained between fine particles, such as fine sand, clay, and silt in the case of TMP, and fine sand and silt in the cases of DM-TMP and OH-TMP, which were lower than −0.76 and −0.75, respectively, while high positive correlations (higher than 0.73) were obtained between the desorption of studied compounds and coarse sand.

The obtained results could be because of two different adsorption–desorption mechanisms of the studied compounds. Firstly, a surface sorption interaction of all studied compounds in the particle size consequently plays an important role, and secondly, an interaction between the soil organic matter and the hydroxyl groups of studied metabolites. The high adsorption of metabolites could be explained by the hydroxyl group, which could enhance the stability of the studied molecules on the soil’s surface because of the formation of hydrogen bonds with the functional groups of the organic matter present in the soil [17].

## 3. Materials and Methods

### 3.1. Chemicals and Reagents, Sampling, and Soil Preparation

All chemicals and reagents used are described in the Supplementary Materials. The structures, p*K*_a_, water solubility, and octanol–water partition coefficient (log *K*_ow_) values of each selected compound are presented in Table S2 (Supplementary Material). Three typical Mediterranean soils were selected, which are ubiquitously distributed throughout the Mediterranean region and have different physicochemical characteristics. Soil 1 is present near rivers in the European zone, and it is an alluvial soil; Soils 2 and 3 are distributed in many European countries, such as Italy, Spain, France, Germany, or Greece, and are terra rossa and cambisol soils, respectively. Two kilograms were sampled from each soil and were collected from the surface of the agricultural area (0–20 cm) in Seville (Southwest Spain). The collected soils were freeze-dried in a Cryodos-50 lyophilizer (Telstar, Terrasa, Spain), homogenized with a mortar, sieved (particle size < 2 mm), and kept in glass bottles until sorption experimentation.

### 3.2. Soil Characterization

Soil characterization was performed by texture determination (coarse sand with particle sizes between 2–0.2 mm, fine sand with particle sizes from 0.2 to 0.02 mm, silt with particle sizes in the range from 0.02 to 0.002 mm, and clay with particle size less than 0.002 mm). Electrical conductivity and pH measurements were carried out of a soil:water suspension of 1:2.5 (*w/v*) and organic matter using the Walkley–Black methodology. The selected soils’ physicochemical characteristics are presented in Table 3. The previous presence of TMP and its metabolites in the studied soils was evaluated by the analysis of the selected soils, as reported by Mejías et al. (2022) [37]. In all cases, concentrations of TMP, DM-TMP, and OH-TMP were lower than the method’s detection limits.

### 3.3. Batch Experiments

Sorption experiments were executed in triplicates as per the indications of the OECD guideline 106 [26]. Fifty milliliters of falcon centrifuge tubes were used. All experiments were conducted at 298 K. To judge the degradation of TMP and its metabolites and their potential adsorption into the plastic of the falcon tubes in the assays, a quality control experiment was carried out as a blank experiment without soil. The soil/solution mixture was shaken in a rotator shaker (LLG-uniLOOPMIX2) at 40 rpm. Experiments were performed while keeping all variables constant and changing just one of them. In all experiments, the soil was weighted, 0.01 M of CaCl_2_ aqueous solution was added, and the solid-liquid suspension was shaken for 24 h to pre-equilibrate the soil before the addition of studied compounds.

#### 3.3.1. Soil/Solution Ratio Optimization

The soil/solution ratio was studied to achieve the correct conditions for the experiments. Soil 1, which has an intermedia percentage of organic matter between the three studied soils, was selected. The tested soil/solution ratio was based on previous literature regarding soil adsorption experiments of TMP [1,21,24,25,26]. The amounts of soil (0.1, 0.2, 0.4, and 0.5 g) corresponding to 0.1:1, 0.2:1, 0.4:1, and 0.5:1 of soil/solution ratio (*w/v*) were placed in a falcon centrifuge tube and 0.6 mL of 0.01 M CaCl_2_ solution was added. The soil/solution suspension was shaken for 24 h for soil pre-equilibration. Then, 0.4 mL of standard solution was added to a final concentration of 1 mg L^−1^ of each compound and a final volume of 1 mL. Centrifuge tubes were agitated for 24 h.

#### 3.3.2. Adsorption Kinetic

Adsorption equilibrium time was evaluated to obtain the best conditions for the adsorption experiments. As in Section 3.3.1., Soil 1 was selected for adsorption in this experiment. The soil (0.4 g) was placed in a falcon centrifuge tube, and 0.6 mL of 0.01 M CaCl_2_ aqueous solution was added. The soil/solution mixture was shaken for 24 h for soil pre-equilibration. Then, 0.4 mL of standard solution was added to a final concentration of 1 mg L^−1^ and a final volume of 1 mL. Centrifuge tubes were shaken for 10, 20, 30, 40, 50, 60, 120, 480, and 1440 min. According to the literature on the adsorption of TMP into the soil [1,24,25], 24 h was selected as the final point of kinetic experiments.

#### 3.3.3. Adsorption Isotherms

The adsorption isotherms were evaluated in the three selected soils. For this purpose, 0.4 g portions of each type of soil were placed in a falcon centrifuge tube, and 0.6 mL of 0.01 M CaCl_2_ aqueous solution was added. The mixture of soil/solution was in agitation for 24 h for soil pre-equilibration. Then, 0.4 mL of different standard solutions were added to a final concentration of 0.8, 1.2, 2, 3.2, 4, and 8 mg L^−1^ in a final volume of 1 mL. These concentrations were selected based on previous studies of TMP adsorption in soils [21,24,26]. Centrifuge tubes were shaken for 24 h.

#### 3.3.4. Desorption Isotherms

For the desorption experiments, the soils obtained after adsorption isotherms (using 0.8, 1.2, 2, 3.2, 4, and 8 mg L^−1^ concentrations) were submitted to desorption experiments. For this purpose, 1 mL of 0.01 M CaCl_2_ aqueous solution was added to the soil remaining in the falcon tubes. The mixture was shaken at 40 rpm for 1440 min.

### 3.4. Soil FT-IR Analysis

The soil characterization was carried out using the Fourier transform infrared spectroscopy (FT-IR) method. The FT-IR analysis was conducted with a Cary 630 FT-IR (Agilent, Santa Clara, CA, USA) with attenuated total reflection (ATR). Three selected soils were characterized before and after the adsorption process. The spectral range was between 4000–650 cm^−^^1^, and the spectral resolution was 4 cm^−^^1^.

### 3.5. LC-MS/MS Analysis

After the sorption experiment, the tubes were centrifuged at 5000 rpm for 10 min. The liquid phase was filtered through a 0.22 µm nylon filter. Analysis of TMP and its metabolites was performed by direct injection with liquid chromatography-tandem mass spectrometry (LC-MS/MS). A volume of 10 μL of the filtered sample supernatant was injected into the LC-MS/MS system. Determination was carried out using an Agilent 1290 Infinity II chromatograph (Agilent, Santa Clara, CA, USA). Chromatographic separation was performed using a Zorbax RRHD Eclipse Plus C18 (150 × 3.0 mm i.d., 1.8 μm particle size) column (Agilent, Santa Clara, CA, USA), protected with a Zorbax RRHD Eclipse Plus C18 (3.0 mm i.d., 1.8 µm particle size), guard column (Agilent, Santa Clara, CA, USA), and thermostated at 35 °C. Elution was carried out in an isocratic mode. The mobile phase was composed of 40% 10 mM ammonium formate buffer containing 0.05% of formic acid and 60% of methanol. Elution was carried out at a flow rate of 0.4 mL min^−1^. The total run time was 5 min.

The LC system was coupled to a 6495 triple quadrupole mass spectrometer (MS/MS) equipped with an electrospray ionization source operated in positive mode. The following settings were used: fragmentor, 166 V; capillary voltage, 4000 V; nebulizer pressure, 40 psi; sheath gas flow rate, 12 L min^−1^; sheath gas temperature, 250 °C; drying gas flow rate, 11 L min^−1^; gas temperature, 350 °C. The analyses were carried out using the Multiple Reaction Monitoring mode (MRM). Two transitions were selected for each compound. The most abundant transition (MRM1) was selected for the quantification, while the second transition (MRM2) and the relation between both transitions were used for the identification. Chromatographic and MS/MS conditions can be found in the Supplementary Material (Table S3). The analytical methodology and quality control can be found in the Supplementary Materials as well as the method’s performance (Table S4).

### 3.6. Data and Statistical Analyses

The adsorption and desorption percentages were determined. The sorption kinetic and sorption–desorption isotherms were evaluated by different models. Excel was used to fit all models, and the correlation coefficients (*R*^2^) were applied to evaluate the good fitting of different models to data. All of this data analysis information is widely described in the Supplementary Materials (Tables S6 and S7). Correlation analysis was applied to evaluate the relations between the adsorption and desorption values of the studied compounds and the physicochemical characteristics of the studied soils. Statistical 10.0 software for Windows was used for statistical analysis, and Student’s *t*-test was applied to evaluate the experimental data.

## 4. Conclusions

The mean soil adsorption of TMP and its metabolites was higher than 90.3% for all studied compounds in all selected soils, except for the adsorptions of metabolites of TMP measured in Soil 2 (88.2 and 86.0% for DM-TMP and OH-TMP, respectively) which were significantly different from those measured in Soils 1 and 3 (mean of 94.5 and 92.5% for DM-TMP and OH-TMP, respectively), showing that organic matter positively affects adsorption. In the case of TMP, adsorption does not have a dependence on the soil’s organic matter. These results are confirmed by the adsorption isotherms. Results showed that isotherms were fitted to the Langmuir, Freundlich, and linear models. Two different adsorption mechanisms are shown: First, a surface sorption interaction of all studied compounds in the particle size consequently plays an important role and, second, an interaction between the soil organic matter and the hydroxyl groups of studied metabolites. This group could enhance the stability of the studied molecules on the soil surface because of the formation of the functional groups of the soil organic matter of hydrogen bonds. Adsorption kinetic is better fitted to the PSO model. The adsorption of studied compounds could be involved in different steps. In the first step, external sorption occurs. This process takes place at a high rate due to the abundant active adsorption sites. The second step is where the intraparticle diffusion is rate controlled. Considering the desorption, TMP possesses a higher desorption than its metabolites (mean of 9.0% for TMP, 4.0% for DM-TMP, and 2.0% for OH-TMP). Moreover, Soil 2 (the one with less organic matter content) possesses the highest desorption (mean of 30.9% for Soil 2 than 1.3 and 3.3% for Soils 1 and 3, respectively), confirming previously reported data. This study confirms that TMP and its metabolites present in wastewater can be adsorbed by the soil and then released to the environment, groundwater by leaching, surface water by runoff, or crops, posing potential health and environmental risks. More investigation is needed for the adsorption–desorption mechanisms of emerging pollutants and their degradation products or metabolites.

## Figures and Tables

**Table 1:** Experimental kinetic adsorption parameters isotherms of TMP and its metabolites in soils.

Compound	C_0_ (mg/g)	PFO Model			PSO Model			IPD Model					
		*R* ^2^	*q*_e, cal_ (mg/g)	*k*_1_	*R* ^2^	*k*_2_	*q*_e, cal_ (mg/g)	*R* ^2^_,1_	*k*_ip,1_	C_1_ (mg/g)	*R* ^2^_,2_	*k*_ip,2_	C_2_ (mg/g)
TMP	0.0881	0.9496	7.96 × 10^−5^	0.0041	0.9999	308	0.00197	0.9768	3.0 × 10^−5^	0.0017	0.9821	1.0 × 10^−6^	0.0019
OH-TMP	0.1125	0.4738	9.71 × 10^−5^	0.0039	0.9999	264	0.00206	0.9733	4.0 × 10^−5^	0.0017	0.9288	1.0 × 10^−6^	0.0020
DM-TMP	0.1230	0.8159	2.09 × 10^−4^	0.0027	0.9999	97	0.00203	0.9957	4.0 × 10^−5^	0.0016	0.9853	5.0 × 10^−6^	0.0018

**Table 2:** Experimental adsorption and desorption parameters isotherms of TMP and its metabolites in the soils.

			Adsorption			Desorption		
Soil	Model	Parameter	TMP	DM-TMP	OH-TMP	TMP	DM-TMP	OH-TMP
Soil 1	Linear	*K*_d_ (L/g)	0.014	0.024	0.024	0.024	0.880	-
		*R* ^2^	0.996	0.970	0.970	0.929	0.968	-
	Langmuir	*q*_max_ (mg/g)	0.051	0.076	0.076	0.187	0.044	-
		*K*_L_ (L/g)	0.461	0.560	0.560	0.224	33.51	-
		*R* ^2^	0.993	0.920	0.876	0.908	0.980	-
	Freundlich	*K*_F_ (L/g)	0.016	0.024	0.024	0.056	0.268	-
		*n*	0.778	0.634	0.634	1.320	0.702	-
		*R* ^2^	0.997	0.995	0.979	0.954	0.993	-
Soil 2	Linear	*K*_d_ (L/g)	0.024	0.010	0.010	0.010	0.012	0.007
		*R* ^2^	0.975	0.991	0.991	0.971	0.989	0.955
	Langmuir	*q*_max_ (mg/g)	0.068	0.035	0.024	0.036	0.032	0.031
		*K*_L_ (L/g)	0.537	0.618	9.635	0.404	0.604	0.364
		*R* ^2^	0.992	0.984	0.740	0.974	0.971	0.895
	Freundlich	*K*_F_ (L/g)	0.024	0.023	0.028	0.011	0.023	0.009
		*n*	0.790	0.277	0.489	0.807	0.277	0.904
		*R* ^2^	0.990	0.954	0.721	0.979	0.954	0.896
Soil 3	Linear	*K*_d_ (L/g)	0.007	0.008	0.008	0.019	0.204	-
		*R* ^2^	0.717	0.734	0.734	0.996	0.800	-
	Langmuir	*q*_max_ (mg/g)	0.018	0.018	0.028	0.090	0.018	-
		*K*_L_ (L/g)	5.346	6.782	17.07	0.258	158.4	-
		*R* ^2^	0.857	0.882	0.928	0.987	0.982	-
	Freundlich	*K*_F_ (L/g)	0.013	0.015	0.025	0.019	0.046	-
		*n*	0.368	0.364	0.190	0.909	0.361	-
		*R* ^2^	0.791	0.810	0.833	0.991	0.954	-

**Table 3:** Physical and chemical properties of the studied Mediterranean soils.

	Soil 1	Soil 2	Soil 3
Silt, wt. %	44.5	5.80	18.4
Clay, wt. %	30.8	19.9	16.0
Fine sand, wt. %	16.4	4.70	14.0
Coarse sand, wt. %	8.20	69.5	51.6
Electrical conductivity, mS·cm^−1^	129	75	126
Organic matter, wt. %	0.91	0.58	2.01
CEC, meq kg^−1^	234	200	144
Ca Exchangeable, meq kg^−1^	140	154	106
Mg Exchangeable, meq kg^−1^	26.3	9.5	19.5
K Exchangeable, meq kg^−1^	7.8	2.1	4.6
Na Exchangeable, meq kg^−1^	<0.5	<0.5	<0.5
pH	8.27	8.21	8.06

CEC: cation exchange capacity.

## Data Availability

Not applicable.

## Supplementary Materials

The following supporting information can be downloaded at: https://www.mdpi.com/article/10.3390/molecules28010437/s1, Figure S1: Adsorption percentage of TMP and its metabolites with different soil:solution ratio; Figure S2: Adsorption kinetic fitted to a pseudo-first-order model; Figure S3: Adsorption kinetic fitted to a pseudo-second-order model; Figure S4: Adsorption kinetic fitted to the Weber–Morris model; Figure S5: Adsorption kinetic on the IPD model according to equation *q*_t_/*q*_ref_ vs. t/t_ref_; Table S1: Matrix correlation of TMP and its metabolites adsorption and desorption with soil characteristics; Table S2: Physical and chemical properties of the target compounds; Table S3: LC-MS/MS parameters [38,39,40]; Table S4: Limits of detection (LOD) and quantitation (LOQ), accuracy (%), and precision (expressed as relative standard deviation (*n* = 3)); Table S5: Adsorption and desorption isotherm models studied; Table S6: Adsorption kinetic models studied.

## Sample Availability

Samples of the compounds are available from the authors upon reasonable request.

## References

[1] Franklin A.M., Clinton W., Andrews D.M., Watson J.E. (2022). Sorption and Desorption Behavior of Four Antibiotics at Concentrations Simulating Wastewater Reuse in Agricultural and Forested Soils. Chemosphere.

[2] Gbadegesin L.A., Tang X., Liu C., Cheng J. (2022). Transport of Veterinary Antibiotics in Farmland Soil: Effects of Dissolved Organic Matter. Int. J. Environ. Res. Public Health.

[3] Haenni M., Dagot C., Chesneau O., Bibbal D., Labanowski J., Vialette M., Bouchard D., Martin-Laurent F., Calsat L., Nazaret S. (2022). Environmental contamination in a high-income country (France) by antibiotics, antibiotic-resistant bacteria, and antibiotic resistance genes: Status and possible causes. Environ. Int..

[4] Zhang Y., Cheng D., Xie J., Zhang Y., Wan Y., Zhang Y., Shi X. (2022). Impacts of farmland application of antibiotic-contaminated manures on the occurrence of antibiotic residues and antibiotic resistance genes in soil: A meta-analysis study. Chemosphere.

[5] Harrower J., McNaughtan M., Hunter C., Hough R., Zhang Z., Helwig K. (2021). Chemical Fate and Partitioning Behavior of Antibiotics in the Aquatic Environment—A Review. Environ. Toxicol. Chem..

[6] Kuppusamy S., Kakarla D., Venkateswarlu K., Megharaj M., Yoon Y.E., Lee Y.B. (2018). Veterinary antibiotics (VAs) contamination as a global agro-ecological issue: A critical view. Agric. Ecosyst. Environ..

[7] Mejías C., Martín J., Santos J.L., Aparicio I., Alonso E. (2021). Occurrence of pharmaceuticals and their metabolites in sewage sludge and soil: A review on their distribution and environmental risk assessment. Trends Environ. Anal. Chem..

[8] Santos J.L., Martín J., Mejías C., Aparicio I., Alonso E., Núñez-Delgado A., Arias-Estévez M. (2022). Pharmaceuticals and Their Metabolites in Sewage Sludge and Soils: Distribution and Environmental Risk Assessment. Emerging Pollutants in Sewage Sludge and Soils.

[9] Goldman J.L., Leeder J.S., van Haandel L., Pearce R.E. (2015). In vitro hepatic oxidative biotransformation of trimethoprim. Drug Metab. Dispos..

[10] Zhang C., Barron L., Sturzenbaum S. (2021). The transportation, transformation and (bio)accumulation of pharmaceuticals in the terrestrial ecosystem. Sci. Total Environ..

[11] Wang W.L., Wu Q.Y., Huang N., Xu Z., Lee M.Y., Hu H.Y. (2018). Potential risks from UV/H2O2 oxidation and UV photocatalysis: A review of toxic, assimilable, and sensory-unpleasant transformation products. Water Res..

[12] Carter L.J., Harris E., Williams M., Ryan J.J., Kookana R.S., Boxall A.B.A. (2014). Fate and uptake of pharmaceuticals in soil-plant systems. J. Agric. Food Chem..

[13] Shang H.T., Wang J.L., Wu T., Lin J., Mao B.C. (2020). Adsorption of Naphthalene onto Loess Soil of Northwestern China. Energy Environ..

[14] Zhang J., Zeng J., Mengchang H.E. (2009). Effects of Temperature and Surfactants on Naphthalene and Phenanthrene Sorption by Soil. J. Environ. Sci..

[15] López-Piñeiro A., Peña D., Albarrán A., Becerra D., Sánchez-Llerena J. (2013). Sorption, Leaching and Persistence of Metribuzin in Mediterranean Soils Amended with Olive Mill Waste of Different Degrees of Organic Matter Maturity. J. Environ. Manag..

[16] Sopeña F., Kirk S., Saran S., Gary B. (2012). Assessing the Chemical and Biological Accessibility of the Herbicide Isoproturon in Soil Amended with Biochar. Chemosphere.

[17] Malvar J.L., Santos J.L., Martín J., Aparicio I., Alonso E. (2020). Approach to the Dynamic of Carbamazepine and Its Main Metabolites in Soil Contamination through the Reuse of Wastewater and Sewage Sludge. Molecules.

[18] Martín J., Mejías C., Santos J.L., Aparicio I., Alonso E. (2021). Pharmaceuticals and Their Main Metabolites in Treated Sewage Sludge and Sludge-Amended Soil: Availability and Sorption Behavior. Molecules.

[19] Paz A., Tadmor G., Malchi T., Blotevogel J., Borch T., Polubesova T., Chefetz B. (2016). Fate of Carbamazepine, Its Metabolites, and Lamotrigine in Soils Irrigated with Reclaimed Wastewater: Sorption, Leaching and Plant Uptake. Chemosphere.

[20] Wojsławski J., Białk-Bielińska A., Stepnowski P., Dołżonek J. (2019). Leaching Behavior of Pharmaceuticals and Their Metabolites in the Soil Environment. Chemosphere.

[21] Zhang Y.L., Lin S.S., Dai C.M., Shi L., Zhou X.F. (2014). Sorption-Desorption and Transport of Trimethoprim and Sulfonamide Antibiotics in Agricultural Soil: Effect of Soil Type, Dissolved Organic Matter, and PH. Environ. Sci. Pollut. Res..

[22] Guo X., Wang J. (2019). A General Kinetic Model for Adsorption: Theoretical Analysis and Modeling. J. Mol. Liq..

[23] Xiang L., Wang X.D., Chen X.H., Mo C.H., Li Y.W., Li H., Cai Q.Y., Zhou D.M., Wong M.H., Li Q.X. (2019). Sorption Mechanism, Kinetics, and Isotherms of Di- n-Butyl Phthalate to Different Soil Particle-Size Fractions. J. Agric. Food Chem..

[24] Kočárek M., Kodesova R., Vondrackova L., Golovko O., Fér M., Klement A., Nikodem A., Jaksik O., Grabic R. (2016). Simultaneous Sorption of Four Ionizable Pharmaceuticals in Different Horizons of Three Soil Types. Environ. Pollut..

[25] Lin K., Gan J. (2011). Sorption and Degradation of Wastewater-Associated Non-Steroidal Anti-Inflammatory Drugs and Antibiotics in Soils. Chemosphere.

[26] OECD (2000). Adsorption-Desorption Using a Batch Equilibrium Method. OECD Guideline for the Testing of Chemicals.

[27] Baldermann A., Stamm F.M. (2022). Effect of kinetics, pH, aqueous speciation and presence of ferrihydrite on vanadium (V) uptake by allophanic and smectitic clays. Chem. Geol..

[28] Guo X., Wang J. (2019). Comparison of Linearization Methods for Modeling the Langmuir Adsorption Isotherm. J. Mol. Liq..

[29] Marco-Brown J.L., Guz L., Olivelli M.S., Schampera B., Torres-Sánchez R.M., Curutchet G., Candal R. (2018). New Insights on Crystal Violet Dye Adsorption on Montmorillonite: Kinetics and Surface Complexes Studies. Chem. Eng. J..

[30] Sun Q., Yang L. (2003). The Adsorption of Basic Dyes from Aqueous Solution on Modified Peat-Resin Particle. Water Res..

[31] Wu F.C., Tseng R.L., Juang R.S. (2009). Initial Behavior of Intraparticle Diffusion Model Used in the Description of Adsorption Kinetics. Chem. Eng. J..

[32] Mutavdžić Pavlović D., Tolić Čop K., Barbir V., Gotovuša M., Lukač I., Lozančić A., Runje M. (2022). Sorption of cefdinir, memantine, praziquantel and trimethoprim in sediment and soil samples. Environ. Sci. Pollut. Res..

[33] Mutavdžić Pavlović D., Tolić Čop K., Prskalo H., Runje M. (2022). Influence of Organic Matter on the Sorption of Cefdinir, Memantine and Praziquantel on Different Soil and Sediment Samples. Molecules.

[34] Chen X.-T., Yu P.-F., Xiang L., Zhao H.-M., Li Y.-W., Li H., Zhang X.-Y., Cai Q.-Y., Mo C.-H., Wong M.-H. (2020). Dynamics, thermodynamics, and mechanism of perfluorooctane sulfonate (PFOS) sorption to various soil particle-size fractions of paddy soil. Ecotox. Environ. Saf..

[35] Wang Y., van Zwieten L., Wang H., Wang L., Li R., Qu J., Zhang Y. (2022). Sorption of Pb(II) onto Biochar Is Enhanced through Co-Sorption of Dissolved Organic Matter. Sci. Total Environ..

[36] Rodríguez-López L., Santás-Miguel V., Cela-Dablanca R., Núñez-Delgado A., Álvarez-Rodríguez E., Pérez-Rodríguez P., Arias-Estévez M. (2022). Ciprofloxacin and trimethoprim adsorption/desorption in agricultural soils. Int. J. Environ. Res. Public Health.

[37] Mejías C., Martín J., Santos J.L., Aparicio I., Sánchez M.I., Alonso E. (2022). Development and Validation of a Highly Effective Analytical Method for the Evaluation of the Exposure of Migratory Birds to Antibiotics and Their Metabolites by Faeces Analysis. Anal. Bioanal. Chem..

[38] Chemical BooK Sourcing and Integrating Center of Chemicals Materials in China. https://www.chemicalbook.com/.

[39] DrugBank Online Database for Drug and Drug Target Info. https://go.drugbank.com/.

[40] Lee H.J., Kim D.W., Chung E.G. (2021). Strong links between load and manure and a comprehensive risk assessment of veterinary antibiotics with low KOW in intensive livestock farming watersheds. Chemosphere.

