# Supplementary material for: Thermodynamic and Kinetic Investigation of the Adsorption and Desorption of Trimethoprim and Its Main Metabolites in Mediterranean Crop Soils
Source: Molecules. 2023 Jan 3;28(1):437. doi: 10.3390/molecules28010437 (PMC9823395; doi:10.3390/molecules28010437)
Supplement: Supplementary file 1 [file molecules-28-00437-s001.zip › molecules-2089090-supplementary.pdf]

Supplementary Materials

Thermodynamic and kinetic investigation of the adsorption and desorption of trimethoprim and its main metabolites in Mediterranean crop soils

Carmen Mejías, Juan Luis Santos*, Julia Martín, Irene Aparicio, Esteban Alonso

Departamento de Química Analítica, Escuela Politécnica Superior, Universidad de Sevilla. E-41011 Seville, Spain.

*Corresponding author

Address: Juan Luis Santos Morcillo

Departamento de Química Analítica

Escuela Politécnica Superior

Universidad de Sevilla

c/ Virgen de África, 7

41011 Seville (Spain)

Email address: jlsantos@us.es

Table S1. Matrix correlation of TMP and its metabolites adsorption and desorption with soil characteristics.

	Fine sand, wt. %	Coarse sand, wt. %	Silt, wt. %	Clay, wt. %	pH	EC, mS cm^{-1}	OM, wt. %	CEC, meq kg^{-1}	Ca Exch, meq kg^{-1}	Mg Exch, meq kg^{-1}	K Exch, meq kg^{-1}	TMP (ads)	DM-TMP (ads)	OH-TMP (ads)	TMP (des)	DM-TMP (des)	OH-TMP (des)	Na Exch, meq kg^{-1}
Fine sand, wt. %	**1.00**	**-0.85**	**0.86**	0.45	-0.05	**0.99**	0.52	0.06	-0.58	**0.98**	**0.92**	**-0.68**	**1.00**	**0.90**	**-0.90**	**-0.98**	**-0.98**	0.32
Coarse sand, wt. %		**1.00**	**-1.00**	**-0.86**	-0.49	**-0.76**	0.01	-0.58	0.05	**-0.94**	**-0.99**	**0.97**	**-0.79**	-0.53	**0.99**	**0.74**	**0.73**	0.23
Silt. wt, %			**1.00**	**0.84**	0.46	**0.78**	0.02	0.55	-0.09	**0.95**	**0.99**	**-0.96**	**0.81**	0.56	**-1.00**	**-0.76**	**-0.75**	-0.20
Clay. wt, %				**1.00**	**0.87**	0.31	-0.53	**0.92**	0.47	**0.63**	**0.76**	**-0.96**	0.36	0.02	**-0.80**	-0.28	-0.26	**-0.70**
pH					**1.00**	-0.19	**-0.88**	**0.99**	**0.84**	0.17	0.34	**-0.70**	-0.15	-0.47	-0.40	0.23	0.24	**-0.96**
EC, µS cm^{-1}						**1.00**	**0.64**	-0.09	**-0.69**	**0.93**	**0.86**	-0.56	**1.00**	**0.96**	**-0.82**	**-1.00**	**-1.00**	0.46
OM, wt. %							**1.00**	**-0.82**	**-1.00**	0.32	0.15	0.27	**0.60**	**0.84**	-0.09	**-0.67**	**-0.68**	**0.98**
CEC, meq kg^{-1}								**1.00**	**0.78**	0.27	0.44	**-0.77**	-0.04	-0.38	-0.49	0.12	0.14	**-0.93**
Ca Exch, meq kg^{-1}									**1.00**	-0.39	-0.22	-0.21	**-0.66**	**-0.87**	0.15	**0.71**	**0.73**	**-0.96**
Mg Exch, meq kg^{-1}										**1.00**	**0.98**	**-0.82**	**0.95**	**0.79**	**-0.97**	**-0.92**	**-0.92**	0.11
K Exch, meq kg^{-1}											**1.00**	**-0.91**	**0.88**	**0.66**	**-1.00**	**-0.84**	**-0.83**	-0.07
TMP (ads)												**1.00**	**-0.60**	-0.29	**0.93**	0.53	0.52	0.48
DM-TMP (ads)													**1.00**	**0.94**	**-0.85**	**-1.00**	**-1.00**	0.41
OH-TMP (ads)														**1.00**	**-0.62**	**-0.97**	**-0.97**	**0.70**
TMP (des)															**1.00**	**0.80**	**0.79**	0.13
DM-TMP (des)																**1.00**	**1.00**	-0.49
OH-TMP (des)																	**1.00**	-0.50
Na Exch, meq kg^{-1}																		**1.00**

Exch: exchangeable; des: desorption; ads: adsorption; OM: organic matter; EC: electrical conductivity; CEC: cation exchangeable capacity.

Chemicals and reagents

Standards of high purity of DM-TMP (≥ 98.0%) and OH-TMP (≥ 97.0%) were provided by Toronto Research Chemicals (Toronto, Canada). TMP (≥ 99.5%) was provided from Dr Ehrenstorfer GmbH (Augsburg, Germany). Analytical-grade water, calcium chloride anhydrous ($CaCl_2$) and formic acid were provided by Panreac (Barcelona, Spain). Ammonium formate was supplied by Sigma-Aldrich (Madrid, Spain). LC-MS grade methanol and water were obtained from Biosolve BV (Valkenswaard, the Netherlands). Individual standard solutions of 1000 mg L^{-1} were prepared in methanol and stored in dark in amber glass vials at -18 °C. Standards mixture solutions were prepared at 100 mg L^{-1} in 0.01 M $CaCl_2$ aqueous solution to minimize the methanol co-solvent effect in sorption assays. Other solutions needed were prepared by dilution of standards mixture solution with 0.01 M $CaCl_2$ aqueous solution and stored in amber glass vials at -18 °C.

Table S2. Physical-chemical properties of the target compounds.

Compound	Molecular weight (g mol^{-1})	pK_a	Log K_{ow}	Water solubility (mg/L)	Structure
Trimethoprim (TMP)	290.3	7.16,17.33 [38]	0.91[39]	400 [38]	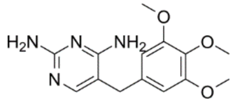
4-hydroxytrimethoprim (OH-TMP)	306.3	8.18 [40]	-	-	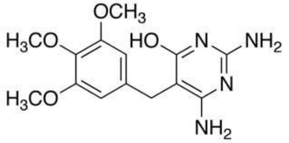
3-desmethyltrimethoprim (DM-TMP)	276.3	9.40 [40]	-	-	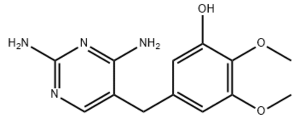

Table S3. LC-MS/MS parameters.

LC-MS/MS parameters						
**Compound**	**Precursor ion *(m/z)***	**Product ions (quantifier/qualifier) *(m/z)***	**Polarity**	**CE (eV)**	**Retention time (min)**	**Ion ratio**
TMP	291.2	229.8/123.0	Positive	28/24	2.056	81.0
OH-TMP	279.2	93.0/121.1	Positive	40/40	1.974	1.0
DM-TMP	277.3	261.4/123.0	Positive	28/44	1.967	63.7

CE: collision energy

Analytical methodology and quality control

The analytical methodology was validated by the determination of the accuracy, precision, and limits of detection and quantification. Accuracy was determined by the injection, by triplicate, of spiked samples at low, medium, and high concentration levels. Precision, measured as relative standard deviation, was determined by the measured, by triplicate, of spiked samples. Limit of detection and quantification, measured as the concentration corresponding to a signal-to-noise ratio of 3 and 10, respectively, were determined by the measured, by triplicate, of matrix-matched standards at low concentration levels. In addition to matrix-matched calibration curves, procedural blanks were injected as quality control.

Table S4. Limits of detection (LOD) and quantitation (LOQ), accuracy (%), and precision (expressed as relative standard deviation (n = 3)).

**Compound**	**LOD (μg L^{-1})**	**LOQ (μg L^{-1})**	**Accuracy (%)**	**Precision (%)**
**TMP**	0.003	0.01	94.7	12
**DM-TMP**	0.015	0.05	89.4	9.5
**OH-TMP**	1.00	3.30	84.1	3.1

Data analysis

The adsorption percentage (%) was calculated, as the difference between the initial concentration of the studied compounds and their final concentration after the adsorption experiment, according to Eq. (1):

Adsorption (%)= $(C_i-C_{e(ads)})/C_i \cdot 100$ (1);

where C_i (mg/L) and $C_{e(ads)}$ (mg/L) are the concentrations of the compound before and after adsorption assay, respectively. The desorption percentage (%) was calculated following Eq. (2):

Desorption (%)= $C_{e(des)}/(C_i-C_{e(ads)}) \cdot 100$ (2);

where $C_{e(des)}$ (mg/L) is the concentration of selected pollutants after desorption experiment.

Eq. (3) was used to calculate the amount of adsorbed emerging pollutant:

$q_{ads}=(C_i-C_{e(ads)}) \cdot V/m$ (3);

where m is the soil mass (g) and V (L) is the final solution volume. Eq. (4) was used to calculate the amount of desorbed pollutant:

$q_{des}=[(C_i-C_{e(ads)})-C_{e(des)}] \cdot V/m$ (4);

A summary of adsorption and desorption isotherms models used is in Table S5 and a summary of adsorption kinetic models can be found in Table S6 in supplementary materials.

Table S5. Adsorption and desorption isotherms models studied.

Isotherm	Model Equation
Langmuir	$q = \frac{q_{max} K_L C_e}{1 + (C_e K_L)}$<br>$C_e$: Equilibrium concentration of pollutants (mg/L);<br>q: Equilibrium sorption capacity (mg/g);<br>q_{max}: maximum amount sorbed within a monolayer (mg/g);<br>K_L: Langmuir dissociation constant (L/g), which is related to the adsorption energy.
Linear	$q = K_d \times C_e$<br>C_e: Equilibrium concentration of pollutants (mg/L);<br>q: Equilibrium sorption capacity (mg/g);<br>K_d: solution-soil distribution coefficient (L/g).
Freundlich	$q = K_F C_e^{1/n}$<br>C_e: Equilibrium concentration of pollutants (mg/L);<br>q: Equilibrium sorption capacity (mg/g);<br>K_F: Freundlich constant (L/g), which is related to the affinity of the adsorbent to the adsorbate;<br>$1/n$: dimensionless parameter, which indicates how adsorption varies as a function of the concentration.

Table S6. Adsorption kinetic models studied.

**Kinetic**	**Model equation**
Pseudo-first order (PFO)	$\ln(q_e - q_t) = \ln q_e - k_1 \times t$<br>$q_e$ and q_t: amounts of compounds (mg/g) adsorbed in the soil at equilibrium and at a t time;<br>t: time (min);<br>k_1: PFO kinetic constants.
Pseudo-second order (PSO)	$t/q_t = 1/(k_2 \times q_e^2) + t/q_e$<br>$q_e$ and q_t: amounts of compounds (mg/g) adsorbed in the soil at equilibrium and at a t time;<br>t: time (min);<br>k_2: PSO kinetic constants.
Intra-particle diffusion (IPD)<br>by Weber-Morris model	$q_t = k_{ip} \times t^{1/2} + C_i$<br>$k_{ip}$: intraparticle diffusion rate constant;<br>C_i: constant proportional to the boundary layer thickness (mg/g);<br>q_t: amounts of compounds (mg/g) adsorbed in the soil at a t time;<br>t: time (min).

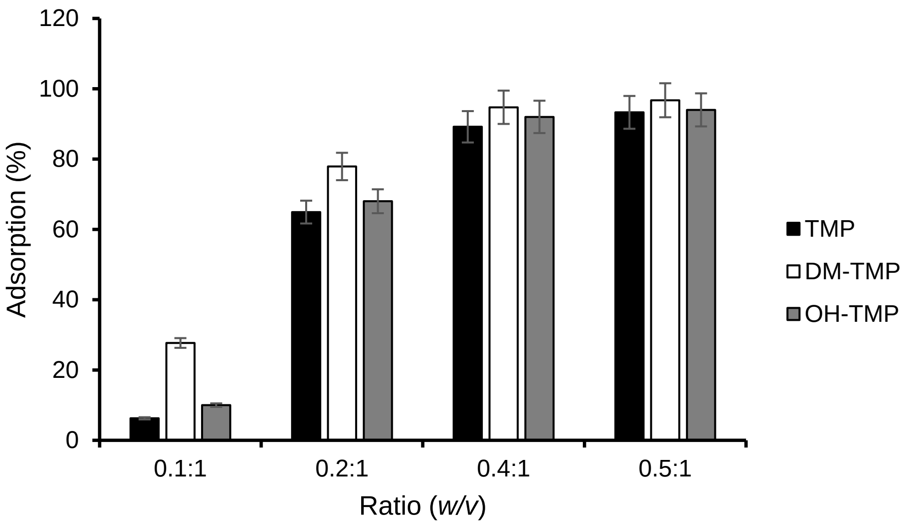

Figure S1. Adsorption percentage of TMP and its metabolites with different soil:solution ratio.

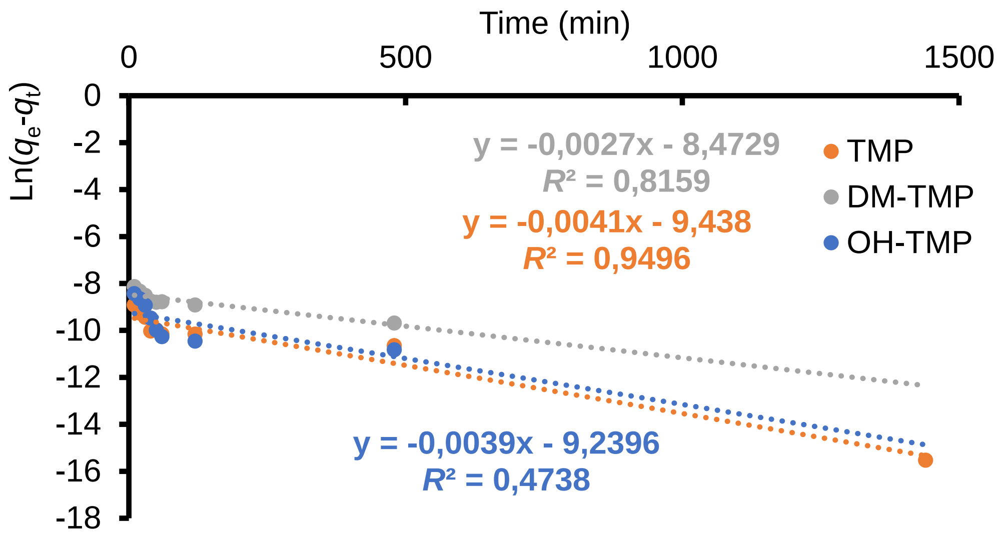

Figure S2. Adsorption kinetic fitted to a pseudo-first order model.

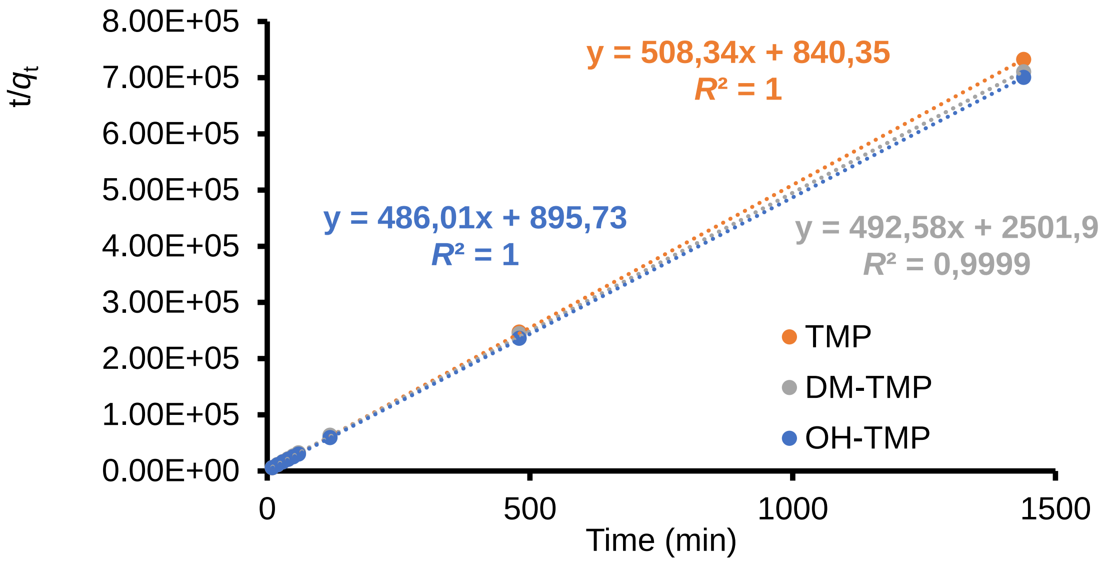

Figure S3. Adsorption kinetic fitted to a pseudo-second order model.

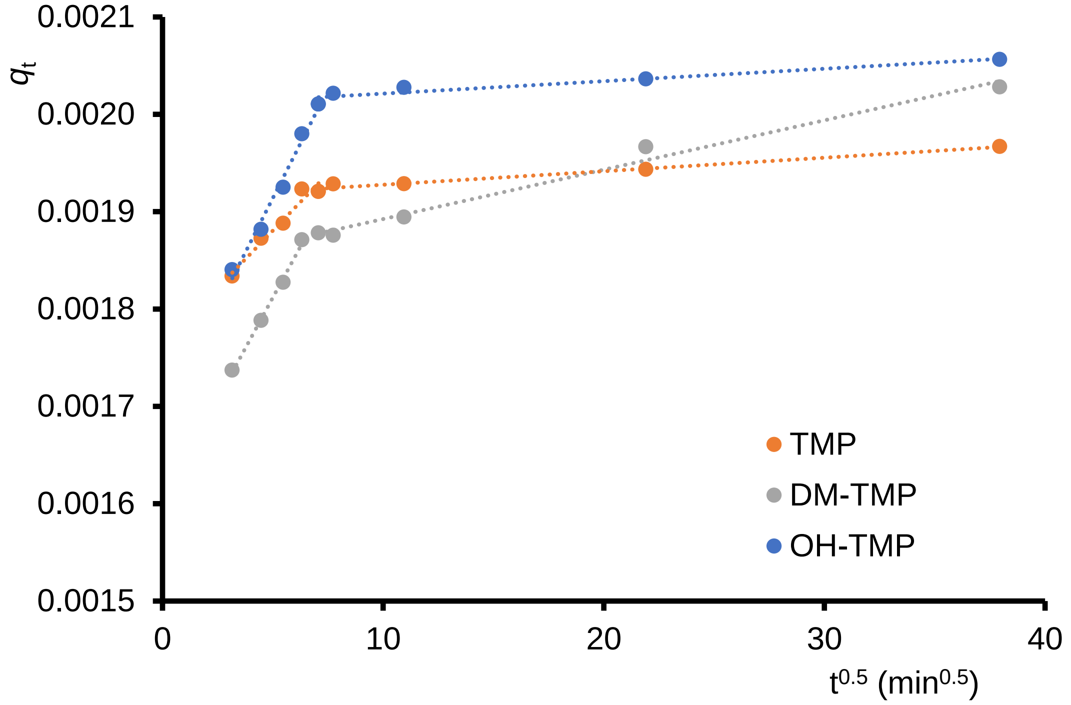

Figure S4. Adsorption kinetic fitted to a Weber-Morris model.

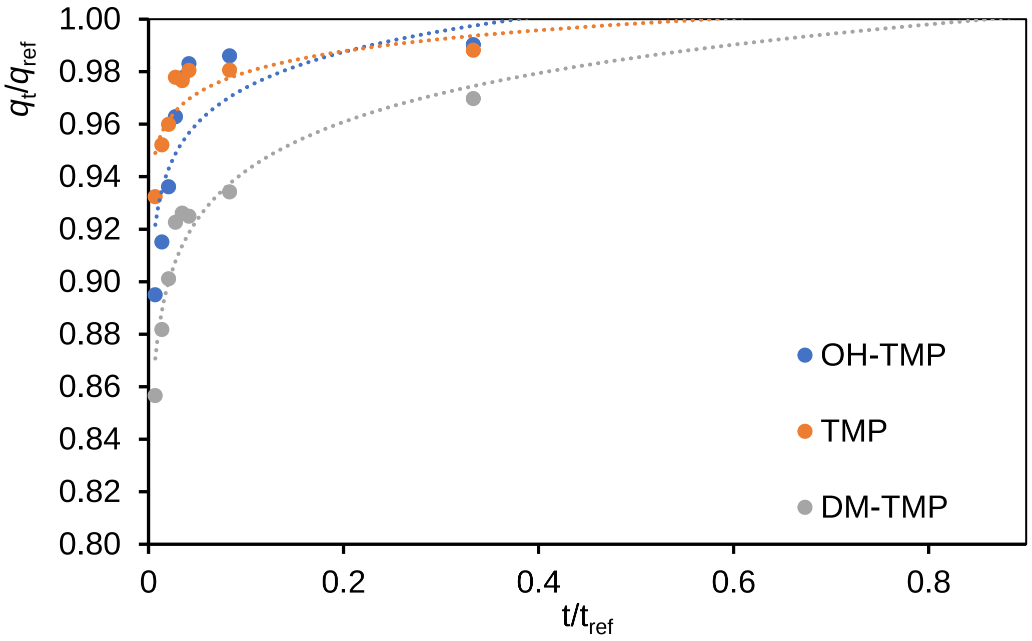

Figure S5. Adsorption kinetic on IPD model according to equation q_t/q_{ref} vs t/t_{ref}.
